# Preliminary Assessment of Health Risks of Potentially Toxic Elements in Settled Dust over Beijing Urban Area

**DOI:** 10.3390/ijerph13050491

**Published:** 2016-05-11

**Authors:** Dejun Wan, Changlin Zhan, Guanglin Yang, Xingqi Liu, Jinsong Yang

**Affiliations:** 1 College of Resource Environment and Tourism, Capital Normal University, Beijing 100048, China; xqliu@niglas.ac.cn; 2 School of Environmental Science and Engineering, Hubei Key Laboratory of Mine Environmental Pollution Control and Remediation, Hubei Polytechnic University, Huangshi 435003, China; chl_zhan@126.com; 3 Institute of Hydrogeology and Environmental Geology, Chinese Academy of Geological Sciences, Shijiazhuang 050061, China; yucb8688@163.com (G.Y.); yjs607@163.com (J.Y.)

**Keywords:** heavy metal, concentration, pollution, enrichment factor, health risk, source

## Abstract

To examine levels, health risks, sources, and spatial distributions of potentially toxic elements in settled dust over Beijing urban area, 62 samples were collected mostly from residential building outdoor surfaces, and their <63 μm fractions were measured for 12 potentially toxic elements. The results show that V, Cr, Mn, Co, Ni, and Ba in dust are from predominantly natural sources, whereas Cu, Zn, As, Cd, Sb, and Pb mostly originate from anthropogenic sources. Exposure to these elements in dust has significant non-cancer risks to children but insignificant to adults. Cancer risks of Cr, Co, Ni, As, and Cd via inhalation and dermal contact are below the threshold of 10^−6^–10^−4^ but As via dust ingestion shows a tolerable risk. The non-cancer risks to children are contributed mainly (75%) by As, Pb, and Sb, and dominantly (92%) via dust ingestion, with relatively higher risks mainly occurring in the eastern and northeastern Beijing urban areas. Although Cd, Zn, and Cu in dust are heavily affected by anthropogenic sources, their health risks are insignificant. Source appointments suggest that coal burning emissions, the dominant source of As, are likely the largest contributors to the health risk, and traffic-related and industrial emissions are also important because they contribute most of the Pb and Sb in dust.

## 1. Introduction

In recent years, outdoor air quality has deteriorated sharply and extremely serious air-pollution incidents occur frequently in China, especially in mega cities in north China [1,2,3,4], posing great threats to human health. The latest model estimation in 2015 suggested that outdoor air pollution, mostly by atmospheric fine particulate matter, leads to about 1.4 million premature deaths per year in China, accounting for 42% of the worldwide total [5]. The main threats of atmospheric particulate matter pollution to human health are associated with exposure to hazardous pollutants adhered on the particles [2,3,5]. Among various hazardous pollutants in atmospheric particulate matter, potentially toxic elements such as Cr, As, Hg, Cd, Sb, and Pb, most of which are heavy metals, are important, because they can bioaccumulate in vital organs in the human body and do harm to circulatory and central and peripheral nervous systems [6,7,8]. Therefore, it is of great significance to choose typical areas/cities in China to investigate health risks of potentially toxic elements in atmospheric particulate matter. 

Among large cities in China, Beijing is not only one of the most densely populated cities, but also one of the most seriously polluted cites for atmospheric particulate matter [1,3,4]. As the capital of China, in recent years, many valuable works have been done to investigate levels, pollution status, temporal variations, sources, causes, and controlling measures of potentially toxic elements in atmospheric particulate matter in Beijing (e.g., [2,9,10,11,12,13,14,15,16]). However, previous studies are often limited by sampling and health risk assessment. Firstly, most previous studies were carried out based on traditional filter membrane sampling method [2,9,10,11,12]. On the one hand, due to the heavy workload of such sampling method, these studies often have difficulty collecting samples from dozens of sampling sites over the entire urban area at the same time, and thus they are often unable to reveal detailed spatial distributions of potentially toxic elements in atmospheric particulate matter. On the other hand, till now there are few such previous studies involving health risk assessment of potentially toxic elements in atmospheric particulate matter. Even if they do, they are only relevant for inhalation exposure and are unable to examine health risks via ingestion and dermal contact, which are often important [7,13]. Additionally, some other studies investigated characteristics and health risks of potentially toxic elements in street dust or foliar dust [13,14,15,16,17]. Such investigators have found it relatively easy collect dust samples from dozens of sampling sites over the entire urban area, but the two kind dusts are often diluted by rain and heavily affected by traffic emissions and thus they may not be a good reflection of the actual risks of potentially toxic elements in atmospheric particulate matter residents are exposed to. Therefore, till now there are still few works that reveal characteristics and health risks of potentially toxic elements in atmospheric particulate matter based on high spatial-resolution sampling in the entire urban area in Beijing. 

Settled dust, deposited at locations with a certain height above the ground and being seldom disturbed by rain and human activities, is often regarded as a faithful reflection of characteristics of atmospheric particulate matter (e.g., [18,19,20]) and is much easier to collect relative to atmospheric particulate matter using traditional filter membrane sampling method. Therefore, in this study, we collected settled dust from 62 sites in Beijing urban area, sieved <63 μm fraction of each dust, which equates roughly to the total suspended particulate matter, and detected 12 potentially toxic elements (V, Cr, Mn, Co, Ni, Cu, Zn, As, Cd, Sb, Ba, and Pb) in the fraction in order to reveal levels, health risks, sources, and spatial distributions of potentially toxic elements in settled dust in Beijing urban area. Such study is significant not only for understanding characteristics and health risks of toxic elements in atmospheric particulate matter at a sub-urban scale, but also for establishing reasonable pollution control measures for environmental management and thus for protecting health of the general population in Beijing City. 

## 2. Materials and Methods 

### 2.1. Study Area

**Figure 1:**
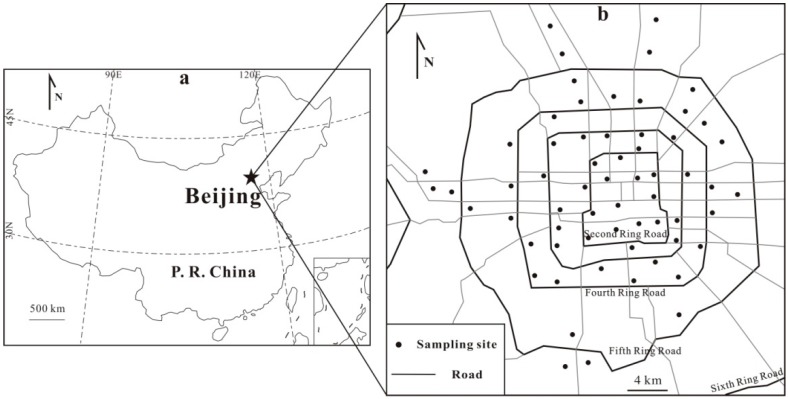
(**a**) Location of the Beijing City in China; and (**b**) the 62 sampling sites for settled dust in the Beijing urban area.

Beijing (39°28′–41°05′ N, 115°24′–117°30′ E), the capital of China, is situated at the northwest of the North China Plain (Figure 1a), with an altitude of 20–60 m above the sea level. It has a temperate continental monsoon climate, characterized by hot and humid summers predominantly influenced by the East Asian monsoon and cold and dry winters predominantly influenced by the vast Siberian anticyclone, with a mean annual temperature of about 11.5 °C and a mean annual precipitation of ~600 mm [18,21]. 

Beijing is one of the largest and most crowded cities in the world. It has a population of 21.5 million (2014), with half of its people living within the Fifth Ring Road (about 710 km^2^) [22]. As the capital of China, in recent years, the government tried to control traditionally heavy-polluting industrial productions in the urban area and most industrial units have migrated to suburban areas or surrounding satellite cities. In contrast, motor vehicles have increased sharply since entering the 21st century. In 2000, the number of motor vehicle was only 1.58 million [23], but by the end of 2014 it had increased to 5.59 million and it is still increasing rapidly [24]. 

### 2.2. Sampling and Analysis

Considering the population in Beijing City is mainly concentrated within the Fifth Ring Road, our sampling work was mainly carried out within this area (Figure 1b) in spring, 2015. Besides, settled dust were also collected in relatively densely populated areas outside the Fifth Ring Road, such as Tiantong Yuan, Shangdi, Shijingshan, Daxing, *etc.* (Figure 1b). At most sites, relatively large residential quarters were chosen for dust sampling. Dust samples were collected using a small brush or a dry-type vacuum cleaner primarily from uncleaned outdoor surfaces of windowsills, flat roofs, pipelines, *etc.* on buildings that are >5 (most >10) m high above the ground and are seldom disturbed by rain and human activities [20,25,26]. The buildings that are far away from main roads, point-source polluting sources, *etc.*, to the extent possible, were chosen for dust sampling. In most sampling sites, dust was collected from several locations of one or two buildings and these sub-samples were mixed into a single composite sample. In total, 62 dust samples were collected (Figure 1b) and stored in coded self-sealing polyethylene bags. 

After being taken back to the laboratory, the dust samples were first sieved through a 250 mesh sieve (pore size ~63 μm). Considering relatively higher health risks of fine particles, only <63 μm fraction was used for element measurement. After being fully dried in an oven at 105 °C for 8–12 h, about 0.125 g ground dust was weighed and then hot-digested with concentrated HNO_3_, HClO_4_, and HCl in Teflon beakers on a hot plate [27,28]. Then, the dust samples were measured for Co, Ni, Cu, As, Cd, Sb, and Pb by an Agilent 7700× inductively coupled plasma mass spectrometry (ICP-MS) and for Al, V, Cr, Mn, Zn, and Ba by a Leeman Labs Profile inductively coupled plasma atomic emission spectrometry (ICP-AES). Blanks and duplicate measurements were performed with the same treatments to ensure quality and accuracy. Replicate analyses suggest that the precision of these determinations is <5% relative standard deviation. Recovery rates for these elements in the international standard reference materials (NBS1645, 1646, and 2704) were about 100% ± 5%.

### 2.3. Enrichment Factor

Enrichment factor (EF) is one of the most commonly used indexes to assess the enrichment degrees and to differentiate sources of elements in environmental media [13,17,29]. The EF value of an element can be calculated as follows:

EF = (X_n_/R)_dust_/(X_n_/R)_background_ (1)

where X_n_ is the concentration of element n and R is the concentration of the reference element. In this study, taking Al as the reference element, two kinds of EF values of these elements in dust were calculated using the local (Beijing) natural soil [30,31] and the upper continental crust (UCC) [32] as the background materials, respectively. 

Based on EF values, the enrichment degrees of elements can be divided into five classes [29] as follows: (1) EF ≤ 2, deficiency to minimal enrichment; (2) 2 < EF ≤ 5, moderate enrichment; (3) 5 < EF ≤ 20, high enrichment; (4) 20 < EF ≤ 40, very high enrichment; and (5) EF > 40, extremely high enrichment.

### 2.4. Health Risk Assessment

In this study, the assessment model of health risk is based on that developed by the US Environmental Protection Agency [33,34]. Human beings are exposed to dust mainly via ingestion, inhalation through mouth and nose, and dermal contact [33]. The non-cancer exposure doses via the three pathways can be estimated by [33,34].

(2) $$D_{\text{ing}}=\text{ C }\times \;\frac{IngR\;\times \;EF\;\times \;ED\;\times \;CF}{BW\;\times \;AT}$$

(3) $$D_{\text{inh}}=\text{ C }\times \;\frac{InhR\;\times \;EF\;\times \;ED}{PEF\;\times \;BW\;\times \;AT}$$

(4) $$D_{\text{derm}}=\text{ C }\times \;\frac{SA\;\times \;SL\;\times ABS\;\times EF\;\times \;ED\;\times EF}{\;BW\;\times \;AT}$$

For cancer risk, the lifetime average daily doses (LADD) (mg·kg^−1^·day^−1^) for Cr, Co, Ni, As, and Cd via inhalation and for As via ingestion and dermal adsorption are estimated by [33,34].

(5) $$LADD=\;\frac{\text{C }\times \text{ EF}}{AT}\;\times \;\left(\frac{CR_{children}\;\times \;ED_{children}}{BW_{children}}\;+\;\frac{CR_{adults}\;\times \;ED_{adults}}{BW_{adults}}\right)$$

where D_ing_, D_inh_, and D_derm_ are the daily exposure doses (mg·kg^−1^·day^−1^) of dust via ingestion, inhalation, and dermal contact, respectively; C (mg·kg^−1^) is the concentration of an element or the upper limit of the 95% confidence interval for the average; IngR is the dust ingestion rate, 200 mg·day^−1^ for children and 100 mg·day^−1^ for adults [7,34,35]; EF is the exposure frequency, 180 day·year^−1^ [25,33,36,37]; ED is the exposure duration, 6 years for children and 24 years for adults [7,33]; BW is the average body weight, 15 kg for children and 60 kg for adults [38]; AT is the averaging time, for non-carcinogens, AT = ED × 365 day; for carcinogens, AT = 70 × 365 = 25550 day [7,33,36]; CF is the conversion factor, 10^−^^6^ kg·mg^−1^ [13,33,34]; InhR is the inhalation rate, 5 m^3^·day^−1^ for children and 20 m^3^·day^−1^ for adults [39]; PEF is the particle emission factor, 1.36 × 10^9^ m^3^·kg^−1^ [25,33,36]; SA is the exposed skin area, 1800 cm^2^ for children and 5000 cm^2^ for adults [39]; SL is the skin adherence factor, 0.2 mg·cm^−2^·day^−1^ for children and 0.07 mg·cm^−2^·day^−1^ for adults [34,37]; ABS, representing an estimate of the relationship between contaminant concentrations in environmental media contacting the skin and the concentrations of these contaminants penetrating into the body, is the dermal absorption factor, 0.001 for elements except As and 0.03 for As [33,34,36]; and CR is the ingestion or inhalation or dermal contact rate (ingestion (CR = IngR × CF), inhalation (CR = InhR/PEF), and dermal contact (CR = SA × SL × ABS × CF)) [33,34,37,38]. 

Hazard quotients (HQ) for non-cancer risks are generated by dividing the exposure daily doses (D_ing_, D_inh_, and D_derm_) of each element with their corresponding reference doses (RfD) (mg·kg^−1^·day^−1^) (Table 1) [33,34,36,37]. Then, the HQs of these three exposure pathways for each element are added to yield a hazard index (HI) to estimate the non-cancer risks of the mixed elements to human beings. HI < 1 indicates no significant risks of the non-cancer effect, and HI > 1 indicates a chance that non-cancer effects may occur, with a probability which tends to increase as the HI value increases [33,36]. Cancer risks of an element are assessed by multiplying its LADD value with its corresponding slope factor (SF, mg·kg^−1^·day^−1^) (Table 1) [33,34,36,40]. SF of As for exposure pathways of ingestion and dermal contact are 1.5 and 3.66, respectively [33,34,37]. The acceptable or tolerable cancer risk for regulatory purposes is in the range of 10^−6^–10^−4^ [33,34].

### 2.5. Principal Component Analysis 

Principal component analysis (PCA) was analyzed using SPSS version 19.0 software (International Business Machines Corporation, Armonk, NY, USA) for windows. It was conducted with varimax normalized rotation. Each principal component (PC) score contains information on all of the metals combined into a single number.

## 3. Results and Discussion

### 3.1. Concentrations of Potentially Toxic Elements in Settled Dust

Statistical description for concentrations of potentially toxic elements in settled dust in the Beijing urban area are given in Table 2 and Table A1. Besides, concentrations of these elements in natural soil in Beijing City [30,31] are also listed. As shown in Table 2, the average concentrations of V, Cr, Mn, Co, Ni, and Ba in dust are generally close (0.7–1.6 times) to their background values in the Beijing natural soil, whereas that of Cu, Zn, As, Cd, Sb, and Pb are often significantly elevated, whose averages are 5.9, 7.0, 3.4, 19.2, 11.2, and 6.6 times higher than that in the local natural soil, respectively. This fact indicates that the former six elements (V, Cr, Mn, Co, Ni, and Ba) in dust are not or only slightly affected by human activities, but the latter six (Cu, Zn, As, Cd, Sb, and Pb) may be affected significantly and thus resulting in high concentrations of these elements in dust. In addition, the coefficient of variations (CV) of the latter six elements are all higher than 35% and basically the highest among these twelve elements (Table 2), further implying that they are affected for different degrees by human activities at different sampling sites.

### 3.2. Enrichment Factors of Potentially Toxic Elements in Settled Dust

**Figure 2:**
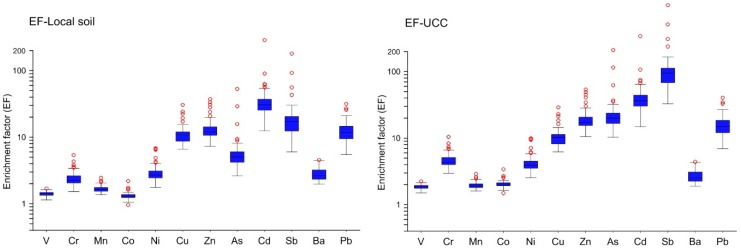
EF values of potentially toxic elements in settled dust in the Beijing urban area relative to the local natural soil and the UCC, respectively.

Taking the local (Beijing) natural soil [30,31] as the background materials (Figure 2), the EF values of V, Cr, Mn, Co, Ni, Cu, Zn, As, Cd, Sb, Ba, and Pb in all the settled dust are on average 1.4, 2.4, 1.7, 1.3, 3.0, 11.3, 13.6, 6.5, 37.1, 21.6, 2.7, and 12.7, respectively. The average EF values of these twelve elements decrease in the following order: Cd > Sb > Zn > Pb > Cu > As > Ni > Ba > Cr > Mn > V > Co (Figure 2).

Taking the upper continental crust (UCC) [32] as the background materials (Figure 2), the EF values of V, Cr, Mn, Co, Ni, Cu, Zn, As, Cd, Sb, Ba, and Pb in all the settled dust are on average 1.9, 4.7, 2.0, 2.1, 4.3, 10.7, 19.6, 25.7, 44.2, 118.5, 2.6, and 16.2, respectively. The average EF values of these twelve elements decrease in the following order: Sb > Cd > As > Zn > Pb > Cu > Cr > Ni > Ba > Co > Mn > V (Figure 2). Compared with the EFs relative to the local soil, the EFs relative to the UCC are obviously higher, especially for Sb and As. The difference is due to the obviously higher concentrations of these elements in the local soil compared to that in the UCC. Concentrations of As and Sb in the Beijing natural soil are about four and five times higher than that in the UCC, respectively. This fact indicates that the EF results relative to the local natural soil as the reference is more significant for assessing pollution levels and for differentiating sources of these elements. Hence, in the following, the EF results relative to the local soil are employed to draw conclusions. 

Among these elements, Cd shows the highest EF value (Figure 2). The EFs of Cd are mostly (67.7%) between 20 and 40 (Table 3), suggesting a very high enrichment level of Cd in most dust samples, according to the classes (%) of enrichment levels in Table 3. Cu, Zn, As, Sb, and Pb are also of relatively high EF values: 95.2% EFs of Cu, 90.3% of Zn, 47.5% of As, 72.6% of Sb, and 93.5% of Pb are between 5 and 20 (Table 3), suggesting that they are highly enriched. The highly enriched levels of these six elements in dust, along with significant variations of their EF values from different sampling sites, suggest that they are mostly originate from anthropogenic sources.

With respect to the other six elements of V, Cr, Mn, Co, Ni, and Ba, their EF values are generally less than 5 (Table 3), and vary insignificantly from different sampling sites, suggesting that these elements in dust are from predominantly natural sources.

### 3.3. Health Risks of Potentially Toxic Elements due to Dust Exposure

Assessment results of health risks, including hazard quotients (HQs), hazard indexes (HIs) and cancer risks, of potentially toxic elements in settled dust in the Beijing urban area are shown in Table 4. From the table it can be seen that the total HI values of non-cancer risks of these twelve elements due to dust exposure are 1.8 and 0.25 to children and adults, respectively, indicating that exposure to these elements in dust has insignificant non-cancer risks to adults but significant risk to children. Health risks of these elements decrease in the order of As > Pb > Sb > Cr > Mn > Ba > V > Cu > Cd > Zn > Ni > Co for children, and As > Pb > Sb > Mn > Cr > Ba > V > Cu > Cd > Zn > Ni > Co for adults. Among the twelve elements, the health risks of As, Pb, and Sb are remarkably higher than the others. These three elements contribute 75% and 74% of the total HIs to children and adults, respectively. Considering the high toxicity of these three elements, attention should be given to As, Pb, and Sb accumulation in children’s body in the Beijing urban area. In conclusion, the health risk results above suggest that potentially toxic elements (especially for As, Pb, and Sb) in settled dust in the Beijing urban area is posing significant threats to children, and thus should be given special attention in future environmental management.

With respect to the three exposure pathways, it can be seen from Table 4 that dust ingestion is the most important for exposure to potentially toxic elements in dust, which contributes 92% and 84% of the total HQs to children and adults, respectively. It is followed by dermal contact, which accounts for 7.4% and 13% of the total HQs to children and adults, respectively. Contributions of the HQs via dust inhalation are the smallest, which are almost negligible to both children and adults. The relative order of HIs for these three exposure pathways is similar to that of dust in other typical cities in China, such as in Huludao [7], Shanghai [35], Xi’an [37], and so on, implying the universality of the importance of dust ingestion to the health risk. 

For cancer risk, the results show that the highest cancer risk 3.8 × 10^−^^5^ is found for As via dust ingestion which falls within the threshold range of 10^−6^–10^−4^, indicating an acceptable or tolerable risk for regulatory purposes and desirable remediation. The cancer risks for the elements of Cr, Co, Ni, As, and Cd via inhalation and dermal contact are all below the acceptable range (Table 4), suggesting that exposure to these elements in dust in the Beijing urban area has no cancer risks to human health via these two exposure pathways. 

However, it should be noted that the above health risk results are calculated based only on the twelve elements in dust. If considering other unanalyzed toxic elements such as Hg, Sn, *etc.*, the actual health risks of toxic elements in dust must be more significant. On the other hand, toxic elements cause damages to human beings involving not only their forms (bioavailability) but also particle sizes of dust. The above risk results calculated by current exposure model are all based on their total concentration of dust particles smaller than 63 µm, so the absorbed doses of the toxic elements via the three exposure pathways may be overestimated due to that bioavailable forms of toxic elements only account for a limited part of the total [3,8] and relatively larger particles may be blocked through the airway clearance mechanism [3,15,25]. Besides, because the settled dust particles may be larger than the household dust and are seldom affected by indoor pollution sources such as furniture, interior decoration, cooking oil fumes, *etc.*, the risk assessment results based on dust collected from building outside surfaces may not be exactly equivalent to that of atmospheric dust for human exposure mainly in household. Therefore, the risk assessment results of potentially toxic elements are still of some uncertainties and thus needs to be further strengthened. 

### 3.4. Spatial Distribution of Health Risks and Sources of Potentially Toxic Elements in Settled Dust

For non-cancer risks, the total HIs of these twelve elements to adults are between 0.12 and 0.75 for the 62 dust samples, which are all smaller than 1, suggesting no non-cancer risks for dust exposure to adult in the entire Beijing urban area. For children, the total non-cancer HIs are between 0.88 and 5.4, with 89% dust samples higher than 1, suggesting significant non-cancer risks for dust exposure to children in most sites of the Beijing urban area. Therefore, in this part, only the spatial distribution of the non-cancer risks for children is discussed.

**Figure 3:**
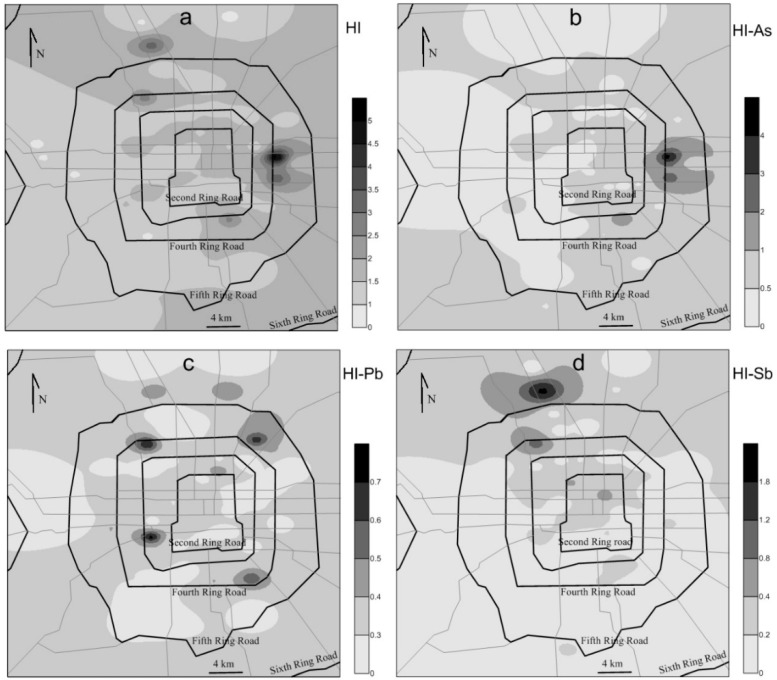
Spatial distribution of HIs (non-cancer risks) for children of: (**a**) the twelve potentially toxic elements; (**b**) As; (**c**) Pb; and (**d**) Sb in settled dust from 62 sites in the Beijing urban area.

Figure 3 shows spatial distributions of the HIs (non-cancer risks) for children of the twelve elements and of As, Pb, and Sb. From Figure 3a, it can be seen that relatively lower health risks of the twelve elements in dust are mainly distributed in the western and southwestern urban areas in Beijing, whereas relatively higher health risks are mainly distributed in the eastern and northeastern.

Among the twelve elements in settled dust, As is the biggest (41%) contributor of the total HIs for children. PCA results suggest that As and Ni show relatively high loading values in PC 5 (Table 5). As and Ni are often regarded as the marker-elements of fly ash of coal burning [3,6], hence PC5 likely reflects the influence of coal burning emissions on the accumulation of As and Ni in dust. Pan *et al.* [2] apportioned sources of 18 trace elements in aerosol collected in Beijing and also found the important contribution of coal burning emissions to As enrichment in aerosols. Considering the biggest (41%) contribution of As to the health risk, this fact implies that coal burning emissions is the most important contributor to the health risk of toxic elements in dust in the Beijing urban area. Based on examining associations of PM_2.5_ exposure from different sources with daily mortality, previous works also suggest that long-term PM_2.5_ exposures from fossil fuel combustion, especially coal burning, but also from diesel traffic, are associated with increased mortality [41,42]. From Figure 3b, it can be seen that relatively higher health risks of As are mainly distributed in the eastern urban areas in Beijing, especially in the center area of the East Fourth Ring Road. Relatively higher risks of As in the Beijing eastern urban areas may due to relatively more coal consumption in Chaoyang District [43]. The highest risks of As in the center area of the East Fourth Ring Road are likely caused by a coal-fired thermal power plant located in this area named Guohua Beijing thermal power plant which was shut down on 20 March 2015 [44]. 

In recent years, the government has tried to improve energy-consuming structure and reduce amount of coal consumed in Beijing City. Although the latest investigation in 2015 suggest that the contribution (22.4%) of coal burning has decreased to the second contributor, smaller than that (31.1%) of vehicle emissions, to atmospheric fine particulate matter among local pollutants [45], coal burning emissions are still the most important factor to elevate health risk of toxic elements in settled dust in Beijing. Therefore, coal burning emissions, especially their related As emission, should be taken further effective measures to control as soon as possible in Beijing City. 

The second most important contributor of health risk for children is Pb, which contributes 19% of the total HI. From Figure 3c, it can be seen that relatively higher HI values of Pb mainly occur in Zhongguancun, the Beijing West Railway Station, Wangjing and the southwestern area of the Fourth Ring Road, all of which are areas with busy and congested traffics. This fact implies that the relatively higher HI values of Pb in dust are likely related to traffic-related pollutions, such as vehicle emissions, wearing of tire and other parts, *etc.* [2,6,10]. PCA results reveal that Pb and Sb and Pb and Cu show relatively high loading values in PC2 and PC3, respectively (Table 5), further indicating the Pb enrichment in dust is affected by not only traffic-related pollutions but also other anthropogenic sources like industrial emissions. 

Antimony contributes 16% of the total HI to children of toxic elements in dust, ranking as the third among the twelve elements. Its spatial distribution pattern shows that relatively higher HIs of Sb occur mainly in the northern part of the Beijing urban area, especially in the northwestern area (Figure 3d). PCA results reveal that Sb and Pb show relatively high loading values in PC2 (Table 5), indicating that Sb enrichment in settled dust is mainly affected by the traffic-related pollution.

For other elements, they show relatively insignificant health risks for children, hence their spatial distributions are not discussed there. With respect to their sources, PCA results (Table 5) suggest that V, Mn, Cr, and Co show high loading values in PC1. These four elements are basically uninfluenced by anthropogenic pollution sources. Therefore, PC1 reflects the predominant influence of natural sources on V, Mn, Cr, and Co in settled dust. In PC4, elements of Ba and Zn show high loading values, implying the influence of waste incineration on dust in the Beijing urban area [6,11]. 

### 3.5. Relationship between Health Risks and Enrichment Factors of Potentially Toxic Elements in Settled Dust

**Figure 4:**
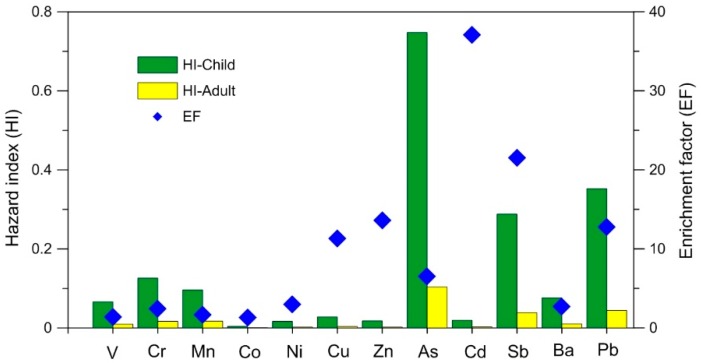
A comparison of health risks for children and adults and EFs of potentially toxic elements in settled dust in the Beijing urban area.

Health risk results suggest that HIs of As, Pb, and Sb are the three biggest contributors of the total HIs for both children and adults, but EF values of these three elements are not the highest, especially for As (Figure 4). Among the EF values of these twelve elements, Cd has the highest EF, but it only contributes ~1% of the total HIs for both children and adults (Figure 4), and Zn and Cu are similar to Cd. On the contrary, the average EF values of V, Cr, and Mn in dust are all extremely low, only 1.4, 2.4, and 1.7, respectively, but they still contribute 15.7% and 17.2% of the total HIs for children and adults, respectively (Figure 4). The phenomenon suggests that the health risks of some heavily polluted elements may be insignificant, for example Cd, Zn, and Cu in dust, whereas the health risks of some slightly or even unpolluted elements may not be negligible, for example V, Cr, and Mn. This may be caused by that the health risk of an element in dust involves not only its pollution level but also absorption, circulation, and other physiological processes in the human body. This fact indicates that pollution assessment of toxic elements in the environmental media may be unable to reflect their environmental risks; hence, health risk assessment is indispensable, especially for environmental media that have relatively close relationships with human beings. 

## 4. Conclusions 

Investigation of 12 potentially toxic elements in the <63 μm fraction of settled dust collected from 62 sites in Beijing urban area has brought out the following conclusions. The average concentrations of V, Cr, Mn, Co, Ni, and Ba in dust are generally close to their background values in the Beijing natural soil, whereas those of Cu, Zn, As, Cd, Sb, and Pb are often significantly elevated. EF results further suggest that V, Cr, Mn, Co, Ni, and Ba in dust are insignificantly influenced by anthropogenic sources, whereas Cu, Zn, As, Cd, Sb, and Pb are often heavily influenced by anthropogenic pollution sources. The average HI values of non-cancer risks of the twelve elements in dust are 1.8 and 0.25 for children and adults, respectively, indicating that exposure to these potentially toxic elements has insignificant non-cancer risks to adults but significant risks to children. Among the three exposure pathways, ingestion is the most important, contributing 92% and 84% of the total HQs for children and adults, respectively. Spatial distribution results suggest that relatively higher health risks for child are mainly distributed in the eastern and northeastern Beijing urban areas, mainly resulting from accumulation of As in dust caused by coal burning emissions in these areas. Besides, traffic-related and industrial emissions are also very important to the health risks because they contribute most of Pb and Sb in dust. There is a tolerable cancer risk for As via dust ingestion and no cancer risks for Cr, Co, Ni, As, and Cd via inhalation and dermal contact due to dust exposure. Compared with EF results, the health risks of some relatively heavily polluted elements, such as Cd, Zn, and Cu, are insignificant, whereas the health risks of some slightly or even unpolluted elements, such as V, Cr, and Mn, are not negligible, suggesting the necessity of health risk assessment for environmental media that have relatively close relationships with human beings.

## Figures and Tables

**Table 1:** The reference doses (RfDs) of potentially toxic elements via ingestion, inhalation through mouth and nose, and dermal contact and slope factors (SF_inh_) of Cr, Co, Ni, As, and Cd via inhalation in this study [33,34,36,37,40].

Element	RfD_ing_	RfD_inh_	RfD_derm_	SF_inh_
	mg·kg^−1^·day^−1^			
V	7.00E−03	7.00E−03	7.00E−05	
Cr	5.00E−03	2.86E−05	2.50E−04	4.20E+01
Mn	4.70E−02	1.40E−05	2.40E−03	
Co	2.00E−02	5.71E−06	1.60E−02	9.80E+00
Ni	2.00E−02	2.06E−02	1.00E−03	8.40E−01
Cu	3.70E−02	4.02E−02	1.90E−03	
Zn	3.00E−01	3.00E−01	6.00E−02	
As	3.00E−04	3.00E−04	1.23E−04	1.51E+01
Cd	1.00E−03	1.00E−03	5.00E−05	6.30E+00
Sb	4.00E−04	4.00E−04	8.00E−06	
Ba	7.00E−02	1.43E−04	4.90E−03	
Pb	3.50E−03	3.52E−03	5.25E−04	

**Table 2:** Descriptive statistics for concentrations of potentially toxic elements in settled dust in the Beijing urban area. Additionally, the average concentrations of these elements in natural soil in Beijing City are also listed.

Item	V	Cr	Mn	Co	Ni	Cu	Zn	As	Cd	Sb	Ba	Pb
	mg·kg^−1^											
Sample Number	62	62	62	62	62	62	62	61	62	62	62	62
Average	57.9	86.0	607.1	10.6	45.2	138.4	722.7	23.9	2.29	12.3	752.3	167.9
Min	36.0	45.0	373.0	6.3	24.0	84.0	382.0	9.0	0.9	4.0	535.0	71.0
Max	73.0	179.0	796.0	16.9	109.0	356.0	2156.0	180.0	18.8	110.1	1054.0	396.0
StDev	7.9	24.8	79.4	1.8	16.1	49.6	335.5	24.3	2.3	14.9	131.1	68.2
Coeff. Variat. (%)	13.6	28.9	13.1	16.7	35.7	35.9	46.4	101.7	100.2	121.3	17.4	40.6
Aver. (Natural soil) ^1^	79.2	68.1	705.0	15.6	29.0	23.6	102.6	7.1	0.119	1.1	531	25.4
Dust/Soil ^2^	0.7	1.3	0.9	0.7	1.6	5.9	7.0	3.4	19.2	11.2	1.4	6.6

^1^ Data of As and Cd in the Beijing natural soil are from Chen *et al.* [31], and that of the other elements are from China National Environmental Monitoring Center (CNEMC) [30]; ^2^ Dust/Soil represents ratios of element concentrations in settled dust to that in the local natural soil.

**Table 3:** Percentages (%) of EFs in each enrichment level for potentially toxic elements in settled dust in the Beijing urban area.

Range	Enrichment Level	V	Cr	Mn	Co	Ni	Cu	Zn	As	Cd	Sb	Ba	Pb
		(%)											
EF ≤ 2	Deficiency to minimal enrichment	100	24.2	95.2	98.4	3.2	-	-	-	-	-	6.5	-
2 < EF ≤ 5	Moderate enrichment	-	74.2	4.8	1.6	92.0	-	-	49.2	-	-	87.1	-
5 < EF ≤ 20	High enrichment	-	1.6	-	-	4.8	95.2	90.3	47.5	9.7	72.6	6.5	93.5
20 < EF ≤ 40	Very high enrichment	-	-	-	-	-	4.8	9.7	3.3	67.7	21.0	-	6.5
EF > 40	Extremely high enrichment	-	-	-	-	-	-	-	-	22.6	6.5	-	-

**Table 4:** Hazard quotients (HQs) and hazard indexes (HIs) of potentially toxic elements in settled dust for children and adults through the three pathways in Beijing urban area. Besides, cancer risks of Cr, Co, Ni, As, and Cd via inhalation are also listed.

Element	HQ_ing_		HQ_inh_		HQ_derm_		HI		Cancer Risk
	Child	Adult	Child	Adult	Child	Adult	Child	Adult	Inhalation
V	5.6E−02	7.0E−03	1.0E−06	1.0E−06	1.0E−02	2.5E−03	6.6E−02	9.5E−03	-
Cr	1.2E−01	1.5E−02	3.9E−04	3.9E−04	4.4E−03	1.1E−03	1.3E−01	1.7E−02	2.0E−07
Mn	8.8E−02	1.1E−02	5.4E−03	5.4E−03	3.1E−03	7.5E−04	9.6E−02	1.7E−02	-
Co	3.6E−03	4.6E−04	2.3E−04	2.3E−04	8.2E−06	2.0E−06	3.9E−03	6.9E−04	5.6E−09
Ni	1.6E−02	2.0E−03	2.9E−07	2.9E−07	5.8E−04	1.4E−04	1.7E−02	2.2E−03	2.1E−09
Cu	2.7E−02	3.4E−03	4.5E−07	4.5E−07	9.4E−04	2.3E−04	2.8E−02	3.6E−03	-
Zn	1.8E−02	2.2E−03	3.3E−07	3.3E−07	1.6E−04	3.9E−05	1.8E−02	2.3E−03	-
As	6.6E−01	8.3E−02	1.2E−05	1.2E−05	8.7E−02	2.1E−02	7.5E−01	1.0E−01	2.4E−08
Cd	1.9E−02	2.4E−03	3.5E−07	3.5E−07	6.8E−04	1.7E−04	2.0E−02	2.5E−03	9.5E-10
Sb	2.6E−01	3.3E−02	4.9E−06	4.9E−06	2.4E−02	5.8E−03	2.9E−01	3.9E−02	-
Ba	7.4E−02	9.2E−03	6.6E−04	6.6E−04	1.9E−03	4.6E−04	7.6E−02	1.0E−02	-
Pb	3.5E−01	4.3E−02	6.4E−06	6.4E−06	4.2E−03	1.0E−03	3.5E−01	4.5E−02	-
Total	1.7E+00	2.1E−01	6.7E−03	6.7E−03	1.4E−01	3.3E−02	1.8E+00	2.5E−01	2.3E−07

**Table 5:** Rotated component matrix for concentrations of potentially toxic elements in settled dust in the Beijing urban area. Loading values higher than 0.5 are shown in bold in each principal component.

Element	Component				
	1	2	3	4	5
V	**0.90**	0.16	0.03	0.08	0.05
Cr	**0.64**	−0.07	0.44	−0.15	−0.02
Mn	**0.88**	0.01	−0.02	0.04	−0.01
Co	**0.56**	0.46	−0.08	0.34	0.29
Ni	0.33	−0.19	0.47	−0.16	**0.53**
Cu	0.02	0.10	**0.88**	0.09	0.07
Zn	0.16	0.12	0.31	**0.73**	−0.06
As	−0.03	0.14	0.01	0.02	**0.92**
Cd	0.02	0.45	−0.01	0.07	0.14
Sb	0.11	**0.76**	−0.01	−0.22	−0.09
Ba	−0.05	−0.14	−0.16	**0.80**	0.03
Pb	−0.01	**0.71**	**0.52**	0.05	−0.08
Initial Eigenvalue	2.45	1.61	1.59	1.41	1.26
Percent of variance (%)	20.42	13.41	13.22	11.78	10.52
Cumulative percent (%)	20.42	33.83	47.04	58.82	69.34

## Abbreviations

The following abbreviations are used in this manuscript:

EF	Enrichment factor
HQ	Hazard quotients
RfD	reference doses
HI	hazard index
PCA	principal component analysis
PC	principal component

## Appendix

**Table A1:** The actual analytical results.

Sample Number	V	Cr	Mn	Co	Ni	Cu	Zn	As	Cd	Sb	Ba	Pb
	mg·kg^−1^											
S-1	53.07	61.42	592.48	10.73	38.81	92.37	636.70	14.14	1.49	9.85	651.81	131.11
S-2	54.08	65.64	526.46	10.31	46.06	355.82	947.13	18.18	1.70	14.30	723.74	396.00
S-3	60.81	131.23	572.43	10.72	64.93	152.10	1086.00	16.03	2.13	10.99	651.17	199.70
S-4	62.98	108.66	600.47	11.31	109.44	334.38	601.18	21.95	2.09	7.83	608.58	170.62
S-5	59.59	90.33	622.74	10.45	71.52	124.09	853.91	19.93	2.84	10.60	907.47	161.07
S-6	35.74	54.79	372.73	6.33	34.69	96.41	501.74	13.23	1.66	5.27	583.23	93.01
S-7	70.60	93.28	796.06	12.59	54.54	119.12	702.92	13.15	0.90	3.99	736.68	93.84
S-8	62.65	78.60	630.16	10.51	34.87	110.39	486.79	19.35	1.91	7.90	640.48	174.87
S-9	68.08	82.60	657.67	14.75	41.31	109.54	707.65	36.13	3.92	12.32	716.07	176.49
S-10	54.14	83.68	581.46	9.90	36.38	106.48	635.63	13.77	1.63	5.95	837.70	114.27
S-11	60.91	79.07	657.91	11.55	41.80	159.42	632.66	19.80	2.16	11.74	685.34	213.32
S-12	56.07	71.89	668.91	12.37	40.12	146.75	1869.75	21.20	2.37	10.17	940.62	206.14
S-13	66.82	98.38	685.96	12.35	48.71	174.67	840.78	63.96	2.78	16.44	784.50	187.82
S-14	60.15	169.80	674.47	11.15	44.87	134.71	649.13	20.28	4.34	9.02	649.02	310.87
S-15	67.25	94.65	643.07	12.95	47.83	106.71	500.34	18.35	1.69	7.83	684.73	112.87
S-16	65.10	86.26	600.29	12.01	45.46	143.39	2156.27	17.52	1.85	9.41	782.38	159.26
S-17	60.76	108.41	785.74	11.49	38.72	125.05	724.27	28.71	2.69	13.80	535.24	197.25
S-18	53.37	85.37	523.34	8.92	49.38	135.95	976.46	12.14	1.19	9.09	562.22	89.95
S-19	55.87	67.86	551.92	12.56	40.84	117.14	676.47	23.54	1.71	8.64	1003.12	138.90
S-20	62.52	104.77	618.16	10.56	54.66	145.94	597.82	15.44	1.48	10.63	691.69	190.02
S-21	73.06	147.30	662.53	9.29	44.48	145.79	646.69	16.11	1.72	7.42	623.80	146.56
S-22	59.71	87.42	593.40	11.84	45.46	145.46	649.07	29.84	2.75	13.32	678.34	273.10
S-23	50.52	82.25	577.31	9.48	48.77	121.05	564.52	14.02	1.42	8.29	946.89	104.86
S-24	60.57	73.03	572.74	11.67	50.72	129.92	1009.30	20.70	2.13	12.07	669.42	349.32
S-25	62.77	81.95	680.07	12.15	48.60	134.97	685.57	21.17	2.38	12.95	708.78	232.51
S-26	51.64	101.79	623.47	10.73	54.52	190.74	679.69	15.26	1.71	12.60	730.35	206.07
S-27	67.16	83.89	584.92	11.51	68.76	86.68	431.63	26.01	1.34	5.75	952.62	87.45
S-28	66.37	85.29	596.50	11.75	67.08	92.32	455.10	31.89	1.34	6.19	917.05	96.87
S-29	60.23	80.38	579.06	11.52	44.00	145.38	703.03	23.52	6.14	58.17	684.99	375.11
S-30	66.23	93.52	716.04	11.00	40.38	123.95	590.71	14.92	1.34	8.12	880.25	103.32
S-31	50.73	53.45	519.52	9.42	31.82	92.65	412.70	14.43	1.51	4.76	658.91	90.60
S-32	62.90	86.10	634.65	11.09	46.77	102.59	577.53	16.15	1.15	110.05	592.01	250.95
S-33	56.98	67.09	731.93	7.63	100.84	85.58	463.38	9.48	1.01	5.82	537.07	140.28
S-34	47.98	84.67	523.36	8.95	34.27	96.17	699.51	15.88	1.50	6.14	1053.55	207.30
S-35	58.04	113.27	721.10	8.92	34.21	113.60	550.90	13.43	1.29	5.37	734.30	117.14
S-36	49.20	61.38	600.45	9.40	29.53	117.18	476.43	12.27	1.43	4.57	676.20	117.33
S-37	44.64	58.91	659.14	8.47	29.53	131.60	493.93	12.25	1.54	4.63	676.36	106.56
S-38	50.65	58.33	518.81	8.13	33.25	118.31	429.21	13.54	1.74	4.57	684.22	92.21
S-39	49.12	62.21	473.55	8.29	31.99	159.23	605.64	16.69	2.17	7.48	752.33	214.23
S-40	45.06	85.96	585.88	9.42	35.32	204.41	1288.93	18.00	1.95	6.64	1052.36	125.26
S-41	71.27	100.25	701.74	12.81	45.52	161.20	1390.29	20.37	1.89	12.30	971.06	200.91
S-42	38.10	45.23	396.51	6.65	23.73	83.53	417.94	13.33	1.29	5.87	723.58	154.70
S-43	57.53	80.05	621.66	12.43	38.35	126.25	597.46	No.	1.54	16.11	832.83	180.09
S-44	62.72	82.13	601.23	11.67	43.08	183.64	680.64	31.56	2.42	25.96	714.40	198.17
S-45	60.98	77.07	667.00	11.03	40.63	164.35	676.66	19.27	2.27	14.99	769.04	131.82
S-46	68.91	86.36	633.93	12.40	43.65	157.30	799.48	26.29	2.51	36.34	792.27	164.41
S-47	61.76	83.21	595.00	11.01	44.31	142.98	851.59	22.36	1.92	11.48	807.95	124.12
S-48	55.93	179.17	631.54	10.53	54.31	261.93	537.66	27.47	2.70	7.66	621.02	150.76
S-49	58.50	85.62	576.34	10.73	38.70	133.50	614.87	17.13	1.71	10.77	906.18	253.56
S-50	62.65	79.82	634.60	8.57	26.06	87.58	382.02	12.31	0.89	4.47	555.61	70.85
S-51	65.01	82.45	660.00	10.43	39.43	156.90	598.52	33.11	1.84	11.95	899.02	215.39
S-52	57.50	75.80	563.33	9.59	36.64	97.20	637.88	18.33	2.29	7.27	914.59	111.64
S-53	54.04	89.47	597.62	9.23	92.64	155.03	805.27	180.43	2.44	10.66	765.47	164.39
S-54	57.70	128.06	614.34	10.11	37.80	118.24	527.59	21.78	2.09	10.32	686.71	160.36
S-55	51.63	72.14	534.72	16.90	31.05	109.15	582.88	102.72	3.19	11.42	604.31	147.68
S-56	49.34	64.30	593.94	9.86	33.45	98.19	666.24	18.13	1.84	7.86	969.88	118.53
S-57	57.32	88.74	666.68	11.45	41.51	144.85	640.90	18.09	18.79	8.84	806.16	171.31
S-58	48.12	56.71	520.86	9.07	28.55	108.37	548.10	19.86	1.87	6.64	824.02	138.95
S-59	50.91	65.04	501.12	9.02	32.65	124.68	553.64	16.59	1.95	7.53	684.81	151.77
S-60	59.92	93.35	667.42	11.27	38.62	142.95	710.53	18.87	1.74	11.44	765.35	137.90
S-61	44.92	65.35	458.55	9.67	36.90	158.15	638.01	22.77	2.97	9.15	641.79	142.93
S-62	64.70	94.12	684.96	11.26	45.30	140.76	1749.96	19.07	1.76	12.80	802.59	164.88
